# Draft Genome Sequence of an *Enterococcus thailandicus* Strain Isolated from Bovine Feces

**DOI:** 10.1128/genomeA.00576-16

**Published:** 2016-07-21

**Authors:** Alicia G. Beukers, Rahat Zaheer, Noriko Goji, Shaun R. Cook, Kingsley K. Amoako, Alexandre V. Chaves, Michael P. Ward, Tim A. McAllister

**Affiliations:** a Faculty of Veterinary Science, School of Life and Environmental Sciences, The University of Sydney, Sydney, NSW, Australia; b Lethbridge Research Centre, Agriculture and Agri-Food Canada, Lethbridge, Alberta, Canada; c Canadian Food Inspection Agency, National Centers for Animal Disease, Lethbridge Laboratory, Lethbridge, Alberta, Canada

## Abstract

Here, we report the first draft genome sequence of *Enterococcus thailandicus* isolated from the feces of feedlot cattle in Southern Alberta.

## GENOME ANNOUNCEMENT

*Enterococcus thailandicus* was first isolated from fermented sausage in Thailand in 2008 (1) and has been identified in the feces of swine (2). We isolated *E. thailandicus* with an intermediate resistance to erythromycin from bovine feces in Alberta, Canada, in 2005 (3). This isolate was originally identified through a previously unobserved variance in the *groES-EL* spacer region (4). The nonexistence/unavailability of an *E. thailandicus* genome sequence in the database provided the motive for selecting this isolate for whole-genome sequencing. The present genome sequence will help provide further insight and understanding of *Enterococcus* genera.

Here, we report the first draft genome sequence of *E. thailandicus*. Genomic DNA was prepared as described by Klima et al. (5). Indexed paired-end libraries were prepared using the Nextera XT DNA sample preparation kit (Illumina, Inc., CA) and paired-end (2 × 300 bp reads) sequenced on an Illumina MiSeq platform (Illumina) to yield a total of 1,169,142 reads. High-quality reads were *de novo* assembled using SPAdes version 3.6.0 software (6).

The draft genome of *E. thailandicus* has a total size of 2,603,691 bp with a GC content of 36.7% and consists of 17 contigs ranging from 998 bp to 431,427 bp with an average coverage of 39× and an *N*_50_ length of 337,578 bp. Genome annotation was performed by use of the NCBI Prokaryotic Genome Annotation Pipeline (http://www.ncbi.nlm.nih.gov/genome/annotation_prok/), leading to the prediction of 2,397 protein-coding genes, 56 tRNAs, 1 transfer-messenger RNA (tmRNA), and 5 rRNA operons. At least four multidrug efflux pump proteins were annotated in the genome and may have contributed to the observed intermediate resistance to erythromycin (3). A glycopeptide resistance protein with homology to VanZ was also identified in the genome. VanZ is known to confer low-level resistance to teicoplanin in *Enterococcus faecium* but not to vancomycin (7). No resistance determinants were identified using the Comprehensive Antibiotic Resistance Database (CARDs) (8) or the ResFinder version 2.1 server (9). No virulence factors were identified using the VirulenceFinder version 1.5 server (10). Limitations of databases for both antibiotic resistance and virulence genes could have resulted in unknown resistance or virulence genes remaining unidentified. It is possible that *E. thailandicus* contains further novel antibiotic resistance or virulence genes with further studies required to elucidate this.

The genome was ordered based on alignment against *E. faecium* T110 (accession number CP006030.1) using progressive Mauve (11) and analyzed for the presence of prophage using PHAST (12). Three incomplete and one questionable prophage were predicted in the genome. Six confirmed clustered regularly interspaced short palindromic repeat (CRISPR) arrays were identified using CRISPRfinder (13). Only one CRISPR array was linked to CRISPR-associated (*cas*) genes, consisting of *cas9*, *ca*s*2*, *cas1* and *csn2* and classifying this array as a type II-A system (14). Gene clusters encoding the production of a putative lantipeptide and a bacteriocin were predicted using the Antibiotics and Secondary Metabolite Analysis Shell (15).

The addition of the draft genome of *E. thailandicus* has expanded on the current *Enterococcus* genome database and will be a valuable addition in comparative genomic analysis studies to further understanding of the diversity of the genus *Enterococcus*.

### Nucleotide sequence accession numbers.

This whole-genome shotgun project has been deposited in DDBJ/ENA/GenBank under the accession LWMN00000000. The version described in this paper is the first version LWMN01000000.
